# Inhibition of phospholipase D2 augments histone deacetylase inhibitor-induced cell death in breast cancer cells

**DOI:** 10.1186/s40659-020-00294-3

**Published:** 2020-10-01

**Authors:** Won Chan Hwang, Dong Woo Kang, Youra Kang, Younghoon Jang, Jung-Ae Kim, Do Sik Min

**Affiliations:** 1 Department of Molecular Biology, College of Natural Science, Pusan National University, Busan, 609-735 South Korea; 2 College of Pharmacy, Yonsei University, 85 Songdogwahak-ro, Yeonsu-gu, Incheon, 21983 South Korea; 3 College of Pharmacy, Yeungnam University, Gyeongsan, 712-749 South Korea; 4 Department of Biology and Chemistry, Changwon National University, Changwon, South Korea

**Keywords:** Phospholipase D2, Histone deacetylase inhibitor, Apoptosis, Angiogenesis, Chemoresistance

## Abstract

**Background:**

Histone deacetylase (HDAC) inhibitors are promising anticancer drugs but their effect on tumor treatment has been disappointing mainly due to the acquisition of HDAC inhibitor resistance. However, the mechanisms underlying such resistance remain unclear.

**Methods:**

In this study, we performed Western blot, q-PCR, and promoter assay to examine the expression of HDAC inhibitor-induced phospholipase D2 (PLD2) in MDA-MB231and MDA-MB435 breast cancer cells. Apoptosis and proliferation were analyzed by flow cytometry. In addition to invasion and migration assay, angiogenesis was further measured using in vitro tube formation and chick embryo chorioallantoic membrane model.

**Results:**

HDAC inhibitors including suberoylanilide hydroxamic acid (SAHA), trichostatin, and apicidin, induce expression of PLD2 in a transcriptional level. SAHA upregulates expression of PLD2 via protein kinase C-ζ in breast cancer cells and increases the enzymatic activity of PLD. The combination treatment of SAHA with PLD2 inhibitor significantly enhances cell death in breast cancer cells. Phosphatidic acid, a product of PLD activity, prevented apoptosis promoted by cotreatment with SAHA and PLD2 inhibitor, suggesting that SAHA-induced PLD2 expression and subsequent activation of PLD2 might confers resistance of breast cancer cells to HDAC inhibitor. The combinational treatment of the drugs significantly suppressed invasion, migration, and angiogenesis, compared with that of either treatment.

**Conclusion:**

These findings provide further insight into elucidating the advantages of combination therapy with HDAC and PLD2 inhibitors over single-agent strategies for the treatment of cancer.

## Background

Resistance of cancers to various chemotherapeutic treatment, is a major obstacle in oncology [1, 2]. Since epigenetic alterations are associated with therapeutic resistance and reversible, they are considered as a promising target for therapeutic intervention. Histone deacetylase (HDAC) inhibitors suppress the deacetylase activity of HDAC, leading to unrestricted histone acetyltransferase (HAT) activity and increase gene expression. Several HDAC inhibitors are in clinical trials both in monotherapy and in combination therapy. Most of the responses using HDAC inhibitors as a monotherapy were observed in hematological cancers with only a few observed in solid tumors. HDAC inhibitors induce cell cycle arrest, activation of apoptotic pathways, induction of autophagy and reactive oxygen species generation [3–6]. However, HDAC inhibitors also show therapeutic resistance via epigenetic alternations, drug efflux and pro-survival mechanisms [7–12]. Thus, modulation of target genes responsible for resistance to HDAC inhibitors can be used for strategies to maximize the efficacy of HDAC inhibitors.

Phospholipase D (PLD) hydrolyzes phospholipid to generate phosphatidic acid (PA), which consequently activates a signaling cascade for cell growth and survival [13–15]. Elevated expression and activity of PLD have been found in many types of human cancer, including breast [16], colon [17], and gastric [18]. Microarray data showed that level of PLD1 was upregulated in HDAC inhibitor-treated cancer cells [19]. In particular, the expression level of PLD2 has been correlated with the survival of patients with colorectal carcinoma [20]. Moreover, *PLD2* point mutations have been detected in patients with breast cancer [21], and cell invasion of highly metastatic cancer cells is dependent on PLD2 [22]. These reports suggest that upregulation of PLD2 is involved in oncogenic signaling and tumorigenesis. In the present study, we show that expression of PLD2 is upregulated by HDAC inhibitors, and confers resistance to HDAC inhibitors in breast cancer cells. Combination therapy with SAHA and PLD2 inhibitor significantly suppressed cell proliferation and angiogenesis and enhanced apoptosis of breast cancer cells, suggesting that combined treatment with these drugs might offer a promising therapeutic approach to the treatment of cancer by overcoming resistance to HDAC inhibitors.

## Results

### HDAC inhibitors upregulate expression of PLD2

**Fig. 1:**
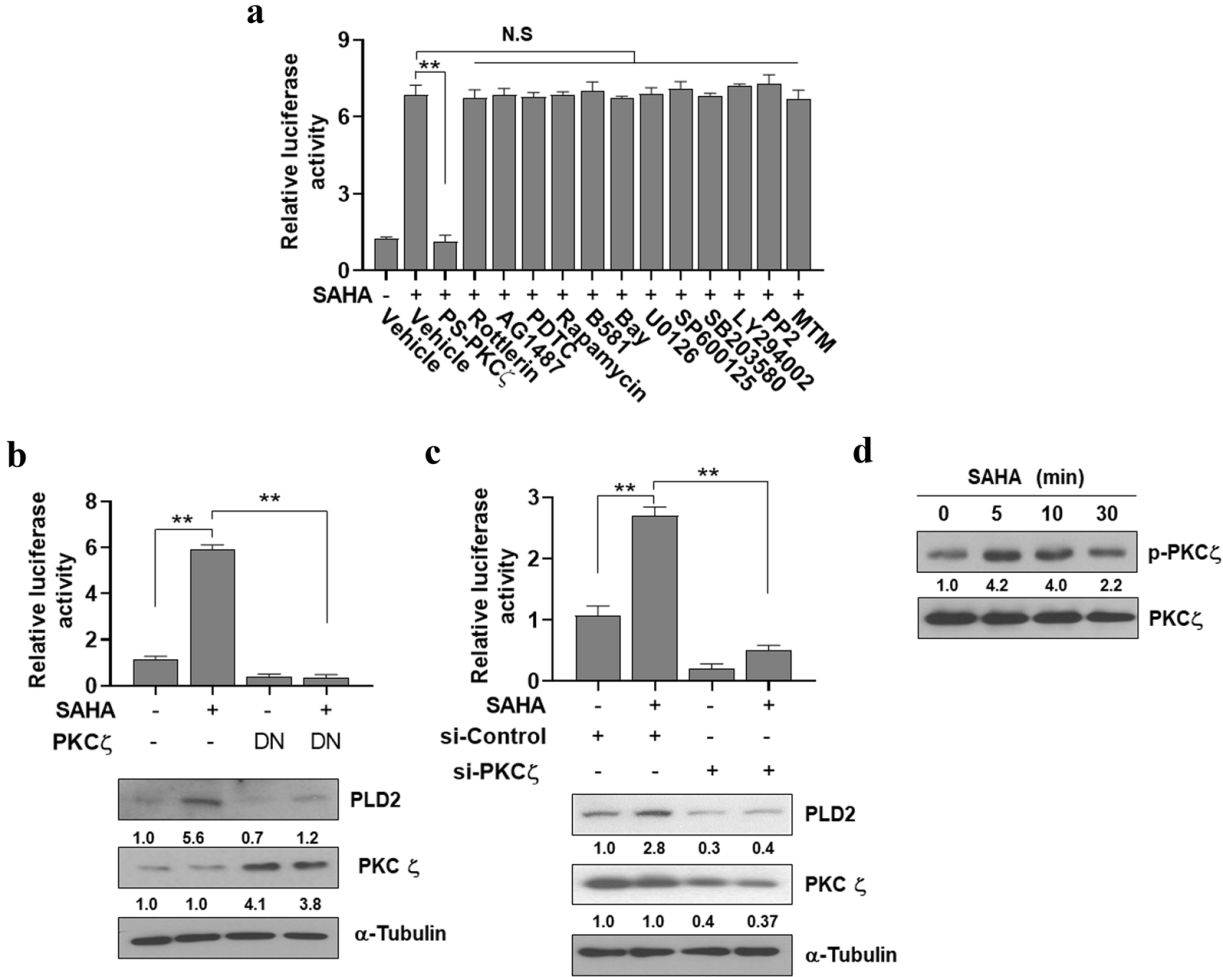
HDAC inhibitors upregulate PLD2 expression in breast cancer cells. **a** The indicated cancer cells were treated with the HDAC inhibitors TSA (400 nM), SAHA (2 μM), and apicidin (5 μM) for 24 h. The lysates were then analyzed by q-PCR and western blot using the antibody to PLD2. **b** MDA-MB 231 cells were cultured and labeled with [^3^H] myristate for 12 h and treated with HDAC inhibitors for 1 h after which PLD activity was measured. **c** MDA-MB 231 cells were treated with the indicated concentrations of SAHA for 24 h or with 2 μM of SAHA for the indicated time, after which PLD2 expression and acetylated histone H4 levels were assessed by western blotting. **d** The cells were transfected with the pGL4-PLD2 promoter and treated with the indicated HDAC inhibitors for 24 h. The level of luciferase activity was then determined. The intensity of the indicated bands was normalized to the intensity of their respective α-tubulin bands and quantified against each other. Results are representative of at least four independent experiments and shown as the mean ± SEM. **p < 0.001 versus vehicle

We investigated whether HDAC inhibitors affect the expression of PLD2. HDAC inhibitors such as trichostatin (TSA), suberoylanilide hydroxamic acid (SAHA, also known as Vorinostat), and apicidin upregulated expressions of PLD2 in MDA-MB 231 and MDA-MB435 breast cancer cells as determined by q-PCR (Fig. 1a). A subtype of breast cancer is basal-like breast cancer, also known as triple-negative breast cancer. Given its lack of estrogen receptor, progesterone receptor, and low expression of human epidermal growth factor receptor, there is no effective biological targeted therapy. MDA-MB231 and MDA-MB435 are known as triple-negative human breast cancer cells, which have highly aggressive behaviors as they go through reattachment, cell metastasis, and cell aggregation. There is a need for an effective therapy that treats triple-negative breast cancer. Moreover, the HDAC inhibitors upregulated the expression of PLD2 protein and increased the level of acetylated histone 4 in the cells, as determined by western blot assay using the antibody to PLD2 (Fig. 1a). Moreover, treatment with the HDAC inhibitors stimulated PLD activity in the MDA-MB 231 cells (Fig. 1b). SAHA, an anticancer drug and the first HDAC inhibitor approved by Food and Drug Administration [23], upregulated PLD2 expression in time- and dose-dependent manners along with increasing the accumulation of acetylated histone 4 in MDA-MB 231 cells (Fig. 1c). All of the tested HDAC inhibitors produced significant increases in promoter activity of PLD2 in the MDA-MB231 and MDA-MB435 cells (Fig. 1d). These results indicate that PLD2 is upregulated by HDAC inhibitors in a transcriptional level.

### PKCζ is required for SAHA-induced PLD2 expression

**Fig. 2:**
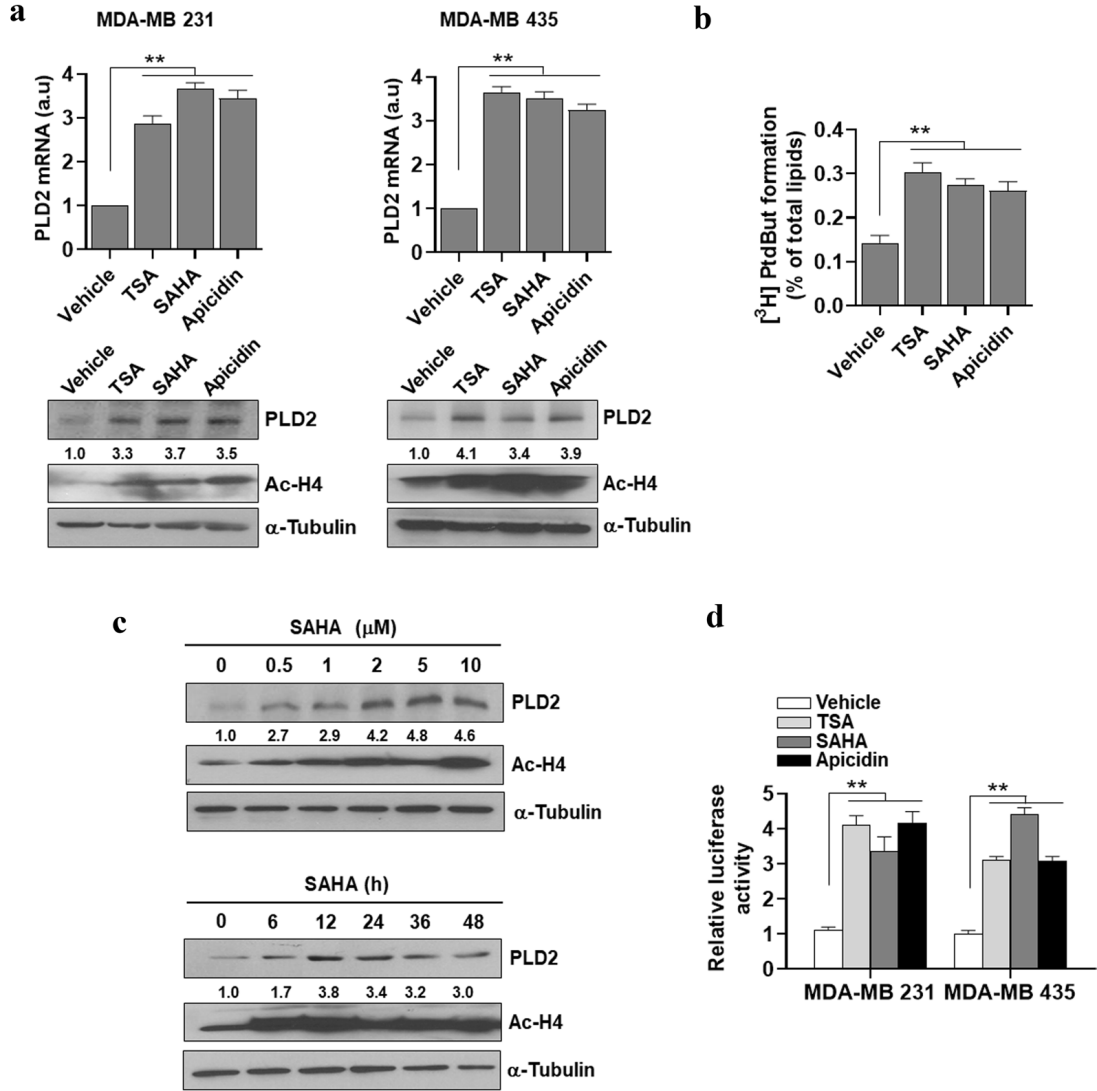
PKCζ is required for SAHA-induced PLD2 upregulation. **a** MDA-MB231 cells were transfected with pGL4-PLD2 and pretreated with various inhibitors, PS-PKCζ (50 μM), Rottlerin (10 μM), AG1487 (10 μM), PDTC (50 μM), rapamycin (10 μM), B581 (50 μM), Bay117085 (5 μM), U0126 (20 μM), SP600125 (50 μM), SB203580 (20 μM), LY294002 (20 μM), PP2 (10 μM), and MTM (5 μM), for 30 min, after which they were treated with SAHA (2 μM) for 15 h. The level of luciferase activity was measured as described in “Materials and methods”. MDA-MB 231 cells were co-transfected with pGL4-PLD2 and DN PKCζ (**b**) or PKCζ siRNA (**c**) and then treated with SAHA for 15 h. Promoter assays and western blotting were performed. **d** MDA-MB 231 cells were treated with SAHA (2 μM) for the indicated time and then analyzed by western blot using the indicated antibodies. The intensity of the indicated bands was normalized to the intensity of their respective α-tubulin bands and quantified against each other. Results are representative of at least four independent experiments and shown as the mean ± SEM. N.S (none significant); **p < 0.001

To investigate whether the certain signaling molecules are required for SAHA-induced PLD2 upregulation, PLD2 promoter activity were measured in MDA-MB231 cells that had been pretreated with various inhibitors prior to incubation with SAHA. SAHA-induced PLD2 expression was largely abolished upon blockade of the activity of the atypical protein kinase C (PKC), PKCζ, by PS-PKCζ (Fig. 2a). Rottlerin (a PKCδ inhibitor), AG1487 (a EGFR tyrosine kinase inhibitor), PDTC (an NF_K_B inhibitor), rapamycin (an mTOR inhibitor), B581 (a Ras farnesylation inhibitor**)**, Bay117085 (an IκBα phosphorylation inhibitor), U0126 (a MEK inhibitor), SP600125 (a JNK inhibitor), SB203580 (a p38 MAPK inhibitor), LY294002 (a PI3K inhibitor), PP2 (an Src inhibitor), and MTM (an Sp1 inhibitor) had no effect on SAHA-induced PLD2 promoter activity (Fig. 2a). As positive controls, efficacy of these inhibitors was confirmed (Additional file 1: Figure S1a, b).

The critical participation of PKCζ in SAHA-induced PLD2 upregulation was confirmed by reporter gene assay and immunoblot analysis (Fig. 2b), which revealed that dominant-negative (DN) PKCζ, a kinase-inactive mutant form of PKCζ, abrogated SAHA-induced PLD2 expression. The role of PKCζ was further investigated by the siRNA analysis in which SAHA-induced PLD2 expression was significantly reduced by knockdown of PKCζ (Fig. 2c). Moreover, SAHA stimulated PKCζ as indicated by the phosphorylation of PKCζ at Thr 410 (Fig. 2d), which is critical for PKCζ activity [24]. Collectively, these results demonstrate that PKC-ζ is critical for SAHA-induced PLD2 expression.

### PLD2 inhibition accelerates SAHA-induced suppression of cell proliferation

**Fig. 3:**
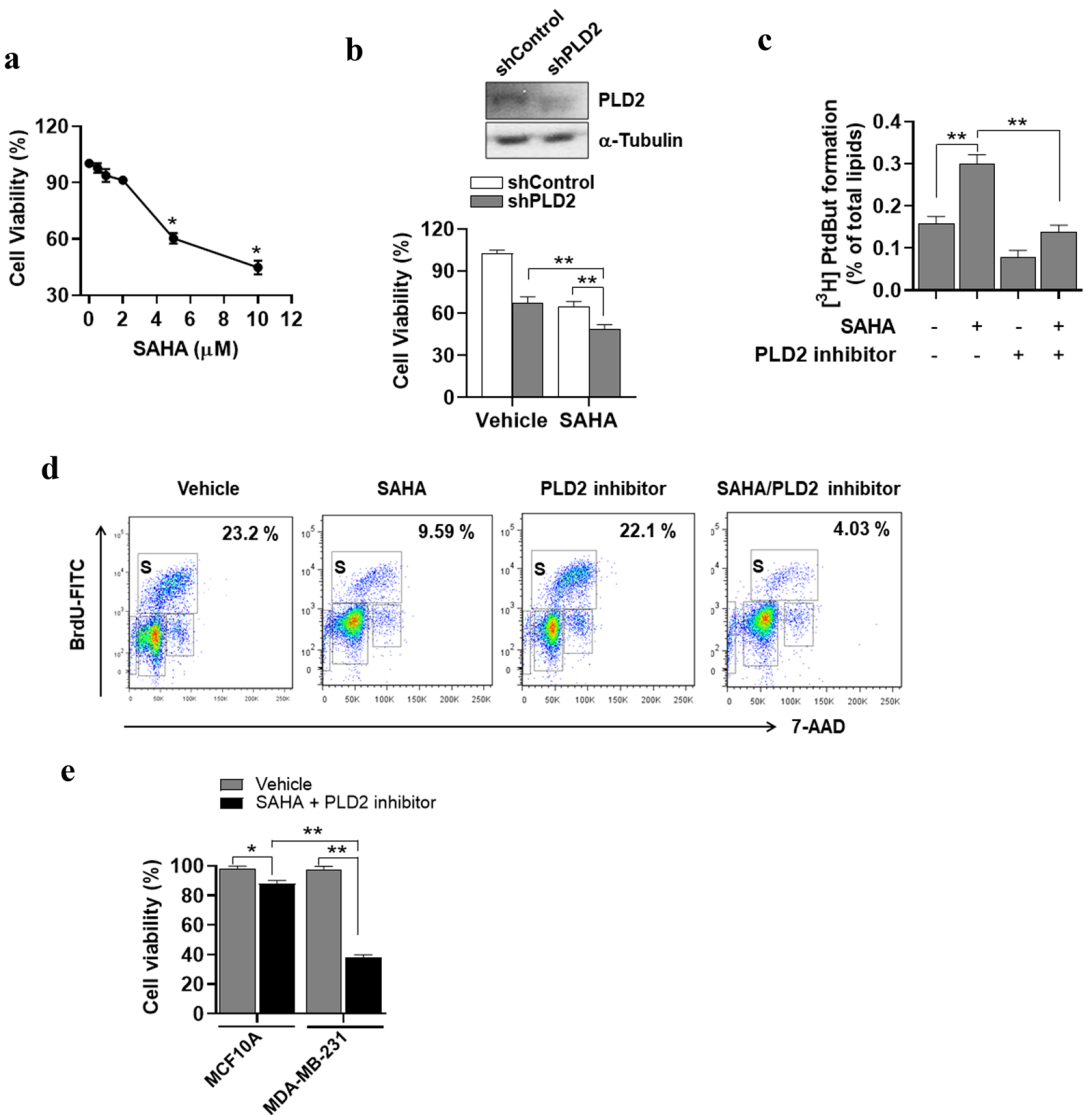
PLD2 inhibition accelerates SAHA-induced suppression of cancer cell proliferation. **a** MDA-MB231 cells were treated with the indicated dose of SAHA for 36 h and cell viability was measured using MTT assay. **b** MDA-MB-231 cells were transfected with siRNA of PLD2 for 24 h and treated with or without SAHA (5 μM) for 36 h. Relative cell viability was examined by MTT assay. **c** MDA-MB-231 cells were treated with SAHA (5 μM) and/or PLD2 inhibitor (10 μM) for 1 h. PLD activity was measured as described in “Materials and methods”. **d** MDA-MB-231 cells were treated with SAHA and/or PLD2 inhibitor for 36 h. The percentage of S-phase cells was determined on the basis of BrdU incorporation in the cells as measured by flow cytometry. **e** MCF10A and MDA-MB231 cells were treated with SAHA (5 μM) and PLD2 inhibitor (10 μM) for 36 h and cell viability was measured using MTT assay. Results are representative of at least four independent experiments and shown as the mean ± SEM. *p < 0.01; **p < 0.001

Next, we investigated whether SAHA affects cell viability. SAHA doses below 2 μM did not affect the viability of MDA-MB231 cell, but SAHA above 5 μM decreased significantly the cell viability (Fig. 3a). Elevated expression and activity of PLD are known to be involved in increased proliferation of cancer cells. Thus, we examined whether SAHA-induced PLD2 expression affects cell viability. PLD2 depletion or SAHA (5 μM) treatment suppressed the viability of MDA-MB231 cells (Fig. 3b). Treatment with 5 μM of SAHA in PLD2-depleted cells significantly reduced cell viability compared to that of either treatment (Fig. 3b). The PLD2-selective inhibitor, VU0285655-1 [25], significantly decreased the SAHA-induced PLD activity as well as the basal PLD activity (Fig. 3c). The BrdU assay with flow cytometry showed that SAHA, but not the PLD2 inhibitor, reduced BrdU incorporation into cells at the S-phase and the combined treatment of SAHA and the PLD2 inhibitor further decreased the BrdU-positive MDA-MB231 cell population in the S-phase compared with the population levels when treated with either treatment separately (Fig. 3d). Furthermore, MCF10A, a normal mammary epithelial cell line, was used to detect the toxicity of the combinational treatment of SAHA and PLD2 inhibitor on normal breast cells. Combined treatment of SAHA and PLD2 inhibitor showed approximately 10% inhibition on MCF-10A cell viability, while the inhibition rate of combined treatment on MDA-MB231 breast cancer cells is approximately 60%, indicating that the combinational effect is more specific for cancer cells (Fig. 3e). These results suggest that inhibition of PLD2 increases SAHA-induced suppression of cell proliferation.

### SAHA-induced PLD2 upregulation increases the threshold for cancer cells to undergo apoptotic cell death

**Fig. 4:**
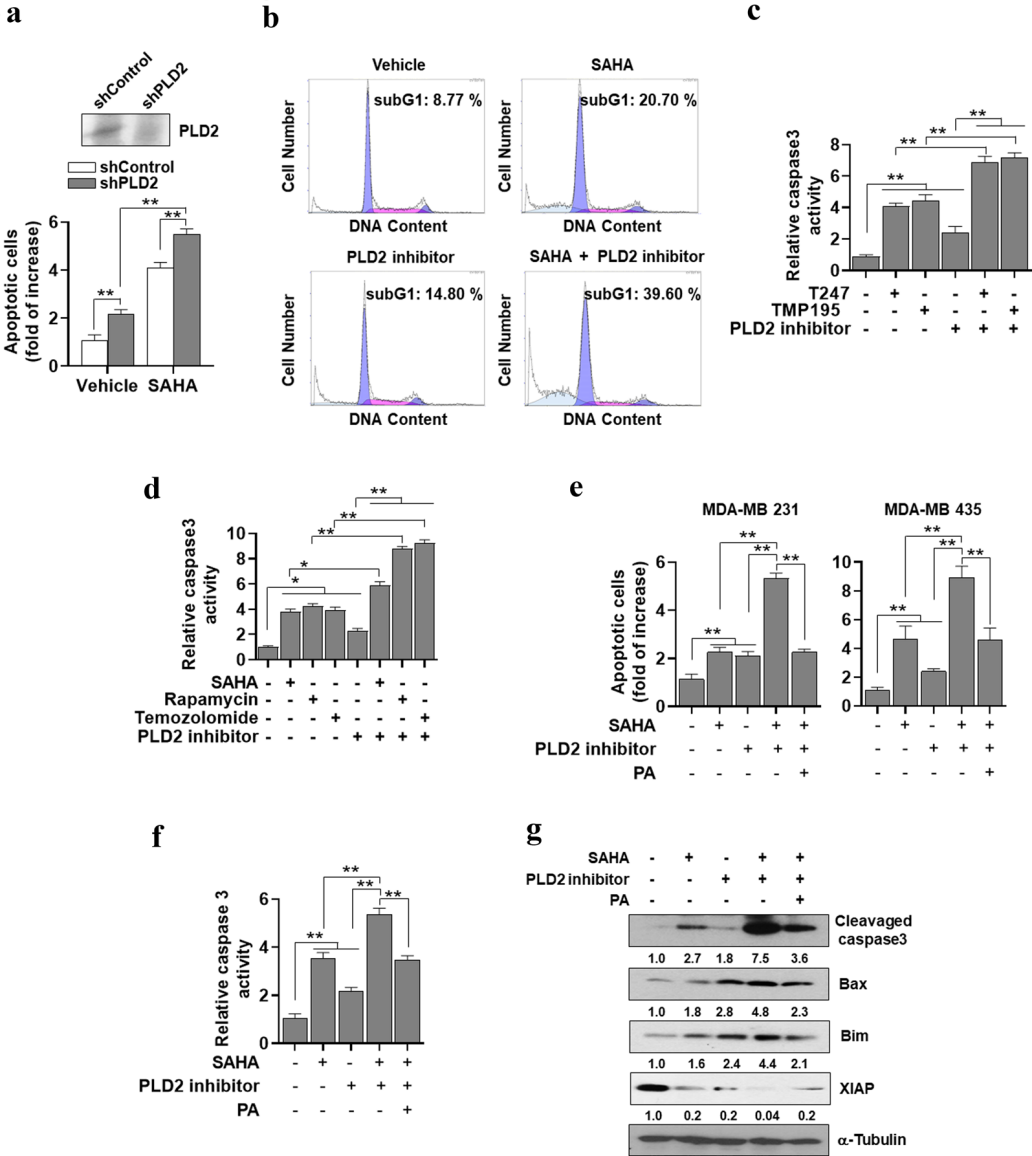
PLD2 inhibition sensitizes SAHA-induced apoptotic cell death. **a** MDA-MB-231 cells were transfected with siRNA of PLD2 and then treated with vehicle or SAHA (5 μM) for 36 h. Apoptosis was examined via flow cytometry and presented as the percentage of cells labeled with annexin V-PE. **b** MDA-MB-231 cells were stained with propidium iodide (1 μg/mL) and the population of subG1 apoptotic cells was determined using FACScan flow cytometry. **c** MDA-MB231 cells were treated with T247 (3 μM), TMP195 (3 μM), and PLD2 inhibitor (10 μM) for 36 h, and caspase-3 activity was examined. **d** MDA-MB231 were treated with SAHA (5 μM), rapamycin (50 nM), temozolomide (50 μM), and PLD2 inhibitor (10 μM) for 36 h, and caspase-3 activity was examined. **e** The cells were treated with SAHA, PLD2 inhibitor, or PA (50 μM) for 36 h. Apoptosis using annexin V staining was examined. **f** MDA-MB-231 cells were treated with the indicated drugs for 36 h, and caspase-3 activity and **g** expression of the indicated proteins were examined. The intensity of the indicated bands was normalized to the intensity of their respective α-tubulin bands and quantified against each other. Results are representative of at least four independent experiments and shown as the mean ± SEM. **p < 0.001

We further investigated whether SAHA and/or PLD2 inhibition affect apoptosis of the cancer cells. Annexin V binding assay showed that SAHA or PLD2 depletion in MDA-MB-231 cells induced apoptosis and PLD2-depletion further promoted SAHA-induced apoptosis in an accumulative manner (Fig. 4a). In addition, MDA-MB-231 cells were stained with propidium iodide and analyzed using flow cytometry. SAHA or PLD2 inhibitor increased the population of subG1 apoptotic cells, while cotreatment further enhanced the population of subG1 cells above that from either treatment (Fig. 4b). Moreover, we investigated the effect of other HDAC inhibitors with different selectivity. T247 is a selective inhibitor of HDAC 3 (class I HDAC) and TMP195 is an inhibitor of HDAC 4 and 5 (class IIa HDAC). Combination of these HDAC inhibitors with PLD2 inhibitor showed accumulative effect on inducing apoptosis of MDA-MB 2312 breast cancer cells (Fig. 4c). In addition, we used non-HDAC inhibitor cytotoxic agents to examine whether the effect of PLD2 inhibitor is restricted to HDAC inhibitor. However, combination of rapamycin or temozolomide, anti-cancer drugs, with PLD2 inhibitor exerted synergistic effects in increasing apoptosis (Fig. 4d). The annexin V binding assay showed that the combined treatment significantly increased apoptotic cell death, compared with that from separate SAHA and PLD2 inhibitor treatments in both MDA-MB-231 and MDA-MB435 cells (Fig. 4e). The enhanced effect of both inhibitors looks like to be accumulation of two different effects that can be mechanistically independent between them. PA, a product of PLD activity, protected against apoptosis induced by SAHA and PLD1 inhibitor (Fig. 4e). Furthermore, the results from caspase 3 activity assay were comparable to those from the apoptosis assay (Fig. 4f). In addition, separate treatments of SAHA or PLD2 inhibitor increased the expression levels of cleaved caspase-3 protein and pro-apoptotic proteins such as Bax and Bim, but decreased the expression of the anti-apoptotic protein XIAP (Fig. 4e), whereas the combined treatment produced further efficacy. PA decreased the SAHA/PLD2 inhibitor-induced expression of active caspase and the pro-apoptotic proteins and recovered the expression of XIAP decreased by cotreatment (Fig. 4e). Taken together, these results suggest that SAH-induced PLD2 upregulation increases the threshold for cancer cells to undergo apoptotic cell death.

### PLD2 inhibition promotes SAHA-induced suppression of invasion, migration, and angiogenesis

**Fig. 5:**
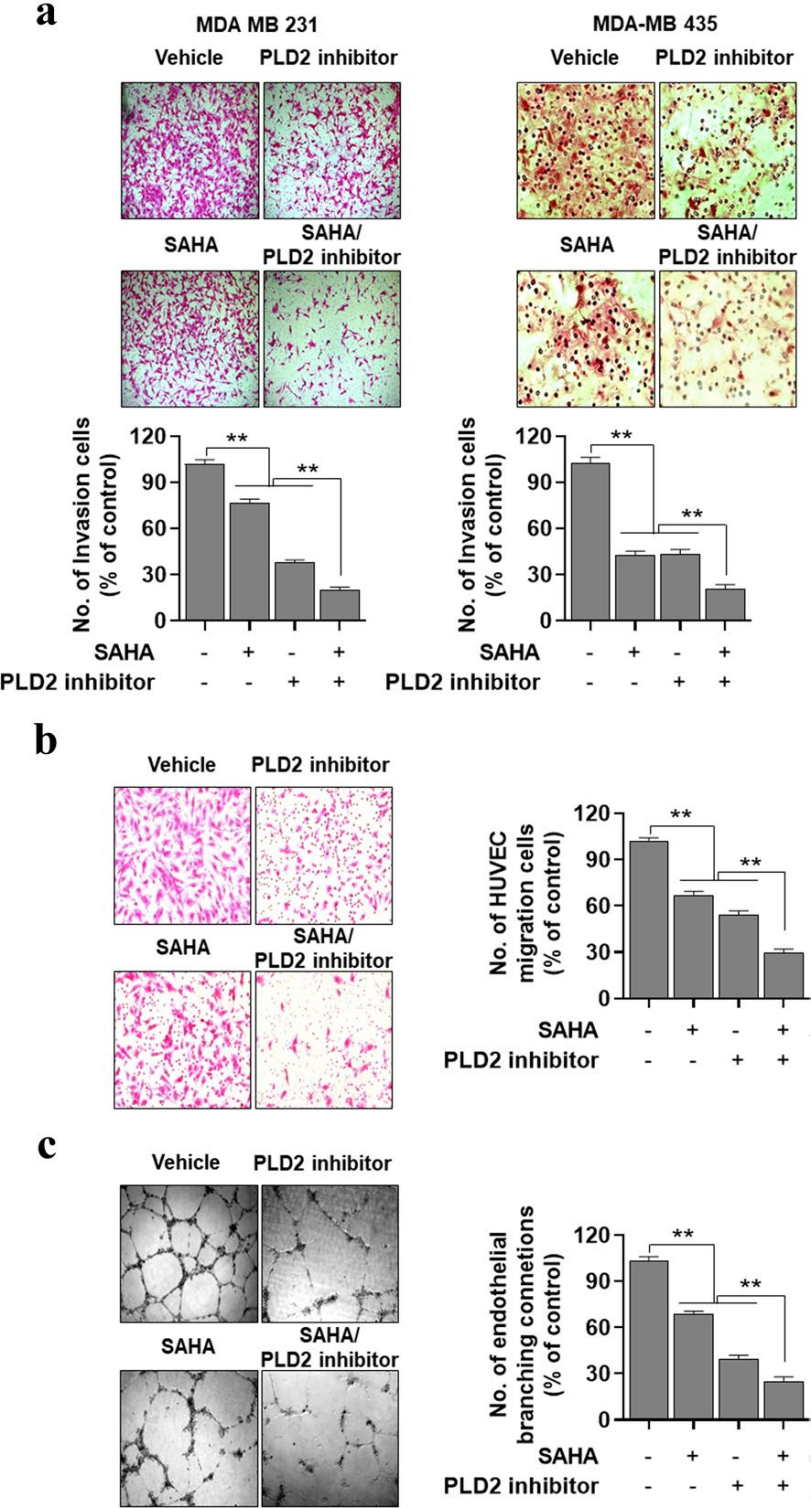
PLD2 inhibitor promotes SAHA-induced suppression of invasion and migration. **a** MDA-MB-231 and MDA-MB435 cells were seeded in Matrigel-coated invasion chambers and then treated with SAHA and/or PLD2 inhibitor for 24 h. The extent of invasion was expressed as an average number of cells per microscopic field. After 24 h, the conditioned medium was collected, applied to HUVECs, and migration **b** and tube formation c were determined. The extent of migration is expressed as the average number of cells per microscopic field. Results are representative of at least four independent experiments and shown as the mean ± SEM. **p < 0.001

**Fig. 6:**
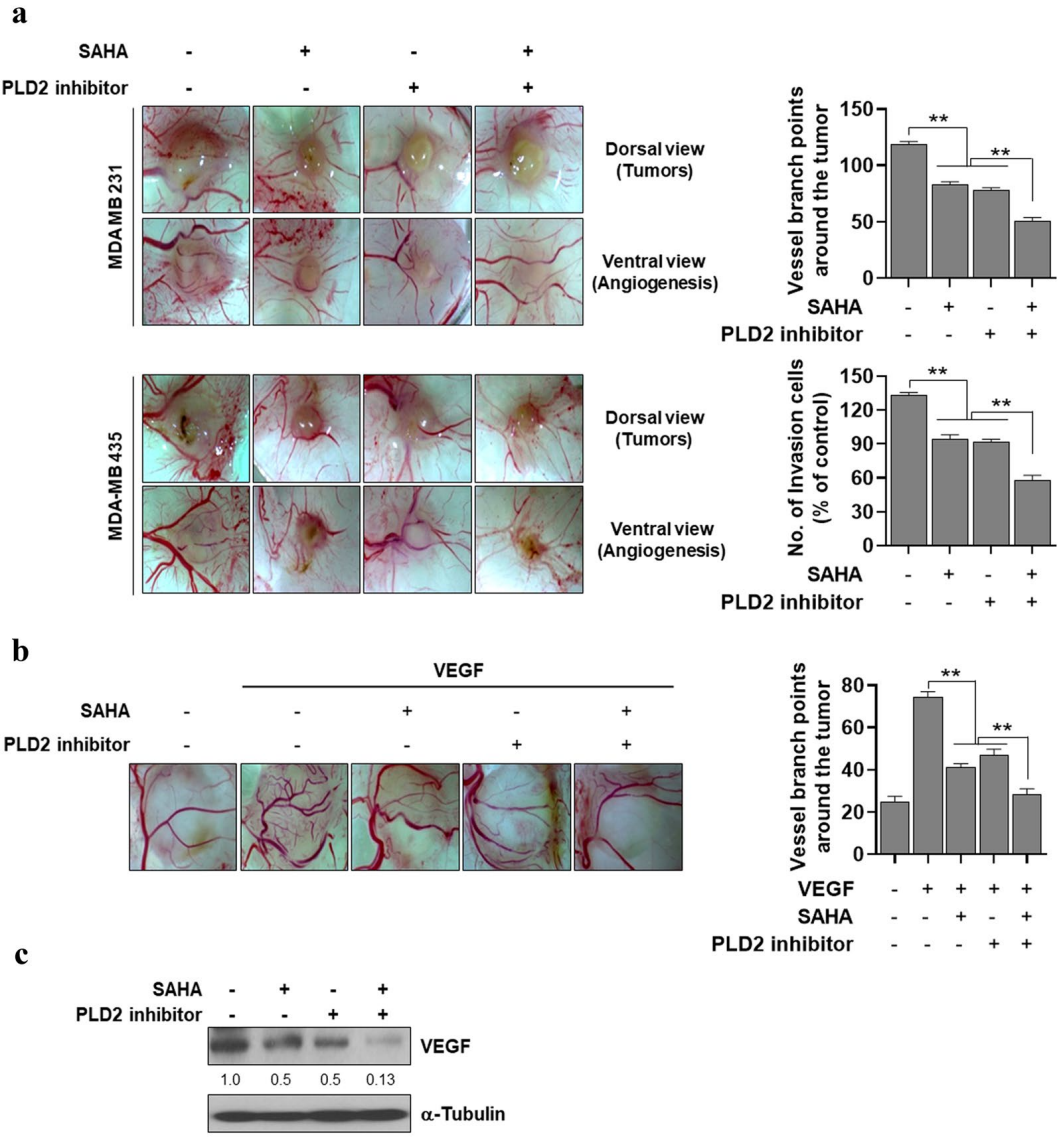
PLD2 inhibitor promotes SAHA-induced suppression of angiogenesis. **a** Inhibitory effects of SAHA and PLD2 inhibitor on angiogenesis in MDA-MB-231 and MDA-MB435 CAM-implanted tumors. The cells were loaded (1.5 × 10^6^ cells/CAM) onto CAMs and SAHA and/or PLD2 inhibitors were administered at the time of implantation. Five days after implantation, the CAM was resected and imaged under the microscope. Tumor vasculature was analyzed and the number of vessels determined. The data shown represent the mean ± SEM of at least six chick embryo CAMs. **b** The CAM of a 10-day-old chick embryo was exposed to vehicle or VEGF. PLD2 inhibitor and SAHA were treated on top of the CAMs by means of filter disks. After a 3-day incubation period, the CAM tissue directly beneath each filter disk was resected from the control and the drug-treated embryo CAMs. Digital images of CAM sections exposed to filters were captured. The data represent the mean ± SEM of at least six chick embryo CAMs. **c** MDA-MB231 cells were treated with SAHA and/or PLD2 inhibitor for 24 h, and the lysates were analyzed by western blot. The intensity of the indicated bands was normalized to the intensity of their respective α-tubulin bands and quantified against each other. Data are representative of three independent experiments. **p < 0.001

We further investigated whether the combined treatment affects invasion, migration, and angiogenesis. Combined treatment with SAHA and PLD2 inhibitor further suppressed the invasive capacity of MDA-MB-231 and MDA-MB435 cells, compared with those from separate treatments of SAHA and PLD2 inhibitor (Fig. 5a). We further investigated the anti-angiogenic effects of the two drugs. The drugs were treated in MDA-MB-231 cells, after which conditioned media were applied to HUVEC prior to undertaking migration and angiogenic assays. PLD2 inhibition significantly enhanced the ability of SAHA to inhibit tube formation and migration, an important feature of angiogenesis (Fig. 5b, c). To further verify the anti-angiogenic and anti-tumorigenic effects of these drugs, we implanted MDA-MB-231 and MDA-MB435 cancer cells into CAMs. The implantation of cancer cells in CAM increased the number of newly formed blood vessels. That tumor-induced neovascularization was significantly suppressed by treatment with PLD2 inhibitor or SAHA (Fig. 6a). Furthermore, the combined treatment further suppressed the amount of neovascularization compared with those from monotherapy (Fig. 6a). Therefore, the potential anticancer efficacy of a SAHA and PLD2 inhibitor regimen is linked to their inhibitory effects against invasion, migration, and angiogenesis. Furthermore, vascular endothelial growth factor (VEGF), which can be used as an in vivo model of angiogenesis, increased the number of newly formed blood vessel branch points in CAM assays (Fig. 6b). The VEGF-mediated neovascularization was suppressed by either treatment. Cotreatment with SAHA and PLD2 inhibitor further suppressed VEGF-induced neovascularization (Fig. 6b). Moreover, the combined treatment of these drugs further inhibited the expression of VEGF in MDA-MB-231 cells, compared with that of either treatment (Fig. 6c). Collectively, the results suggest that PLD2 inhibition significantly promotes SAHA-induced suppression of invasion, migration, and angiogenesis.

## Discussion

The present study demonstrates that PLD2 is upregulated by HDAC inhibitor and PLD1 inhibition increases HDAC inhibitor-induced apoptosis of breast cancer cells. Although HDAC inhibitors have been reported to induce the expression of pro-apoptotic genes, recent studies have demonstrated that they can induce resistance to cancer therapy through the upregulation of urokinase plasminogen activator and *p*-glycoprotein [7, 26]. Thus, to enhance the anticancer activity of HDAC inhibitors, combinational treatments of HDAC inhibitor and various molecular-target drugs [27–30] have been used. Particular research attention has been given to the identification of new targets and elucidating the unknown mechanisms by which HDAC inhibitors may function as effective therapies in cancer treatment. There is accumulating evidence on the critical role played by PLD1 in tumorigenesis and the potential connection of PLD1 with chemoresistance. The fact that PLD and HDAC inhibitors have opposite roles in cancer cells led us to propose that increased PLD expression might enhance the threshold for cancer cells to undergo HDAC inhibitor-induced cell death. Upregulation of PLD1 has been reported in TSA-treated HepG2 cells as analyzed by microarray [19]. Thus, it is suggested that PLD1 might confer resistance to HDAC inhibitors. It has been reported that inhibition of PLD2 has therapeutic potential against cancers [31–34]. Herein, we show that PLD2 is upregulated by HDAC inhibitors in a transcriptional level. SAHA upregulates expression of PLD2 via the PKCζ signaling pathway. Given the inherent resistance to apoptosis, the targeting of alternative pathways is an attractive strategy for improving anti-tumor therapies. SAHA-induced PLD2 upregulation acts as a barrier to apoptotic cell death induced by SAHA. Increased PLD activity due to SAHA-mediated PLD2 expression might be involved in the chemoresistance of HDAC inhibitor because PA prevented apoptosis induced by cotreatment with SAHA and PLD2 inhibitor. Although the effect of combination of HDAC inhibitor with PLD2 inhibitor looks like to be accumulative, the accumulative effects can in fact result in a potential advantage for the potential use of this double treatment in cancer, increasing the spectrum of the targeted pathways. Currently, many researchers are focusing on using a combination strategy involving an HDAC inhibitor with other anticancer drugs [12, 35, 36]. The future design of promising drug combinations will be based on maximizing the potential of HDAC inhibitors and optimizing patient benefit.

## Conclusion

The findings of our study provide evidence that combination of HDAC inhibitor with PLD2 inhibitor may be effective as an anticancer strategy for reducing the resistance to the HDAC inhibitor.

## Materials and methods

### Cell culture and reagents

MDA-MB231 (HTB-260) and MDA-MB435 (HTB-129) cells were maintained in DMEM (SH30243.01, Hyclone, Chicago, IL, USA) medium with 10% fetal bovine serum (SH30084.03, Hyclone) and incubated at 37 °C in a humidified 5% CO_2_ atmosphere (ATCC, Manassas, VA, USA). Normal epithelial cell line MCF-10A cells were cultured in DMEM/F12 supplemented with 20 ng/mL of epidermal growth factor, 100 ng/mL of cholera toxin, 0.01 mg/mL of insulin, 500 ng/mL of hydrocortisone, and 5% horse serum. Human umbilical vein endothelial cells (HUVEC, CRL-1730, ATCC) was maintained in Endothelial Cell Growth Medium (211-500, Sigma Aldrich). Rottlerin (557370), PKCζ pseudosubstrate peptide inhibitor (PS-PKCζ, 539624), B581 (344510), Bay117085 (B5681), PD98059 (513000), and H89 (371963) were from Calbiochem (San Diego, CA, USA). Suberoylanilide hydroxamic acid (SAHA, SML0061), trichostatin A (TSA, T8552), U126 (U120) and apicidin (A8851) were from Sigma Aldrich (St. Louis, MO, USA). T247 and TMP195 were from MedKoo Biosciences (Morrisville, NC, USA). Epidermal growth factor (EGF, 236-EG) and platelet-derived growth factor (PDGF, 120-HD) recombinant proteins were obtained from R&D systems (Minneapolis, MN, USA). The human PLD2 promoter-reporter plasmid (pGL4-PLD2 Luc) has been described elsewhere [37]. pSP1-Luc (LR-2007, Signosis, Sunnyvale, CA, USA). VU0285655-1 (13207) was from Cayman Chemical (Ann Arbor, MI, USA).

### Western blotting

Antibodies against the following proteins were used: PLD2 (sc-515744), BAX (sc-7480), XIAP (sc-55550), Bim (sc-374358), VEGF (sc-7269), EGFR (sc-373746), p-EGFR (sc-81488), AKT (sc-81434), p-AKT (sc-377556), PKCζ (sc-17781), pPKC (sc-12894R), PKCδ (sc-8402), p-PKCδ (sc-377560) and α-tubulin (sc-8035, Santa Cruz Biotechnology, Dallas, TX, USA), acetyl-Histone 4 (06-866, EMD Millipore, Burlington, MA, USA), ERK (#9102), p-ERK (#9101S), JNK (#9252), p-JNK (#4668), IκBα (#4814), p-IκBα (#2859), Src (#2109), p-Src (#2101), S6K (#2708), p-S6K (#9234), p38 (#9212), p-p38 (#9211) and cleavaged caspase 3 (#9661, Cell Signaling, Danver, MA, USA). The signal densities on the blots were measured with Image J (Wayne Rasband) and normalized using anti-α-tubulin antibody.

### q-PCR

q-PCR was performed by using a QuantiTect SYBR green PCR kit (204143, QIAGEN, Hilden, Germany). The q-PCR forward and reverse primer sequences for PLD2 are as follows: forward 5′-CATCCAGGCCATTCTGCAC-3′, reverse 5′- GTGCTTCCGCAGACTCAAGG-3′.

### Luciferase reporter assay

The luciferase activities of the PLD2 promoter were measured using the dual-luciferase reporter assay system (E1910, Promega, Mannheim, Germany) according to the manufacturer’s instructions. Relative luciferase activity was obtained by normalizing the firefly luciferase activity against the renilla luciferase activity.

### Cell viability assay

To assess cell viability, a 3-(4, 5-dimethylthiazol-2-yl)-2, 5-diphenyltetrazolium bromide (M5655, Sigma Aldrich) assay was performed. The percent viability was indicated relative to the control cells.

### Flow cytometry for cell cycle and proliferation assessment

Cells were collected at a density of 2 × 10^6^ cells and fixed with 80% absolute ethanol. After fixation, cells were stained with propidium iodide (1 μg/mL, 81845, Sigma Aldrich) and then analyzed using FACScan flow cytometry. For measurement of cell proliferation, the 5-bromo-2′-deoxyuridine (BrdU, 550891, BD Bioscience, San Jose, USA) cell proliferation assay was performed and proliferating cells were analyzed using flow cytometry.

### Apoptosis assay

Apoptosis was measured by using an annexin V binding assay kit (550474, BD Bioscience) in accordance with the manufacturer’s protocols, after which the annexin V-positive cells were quantified. Hoechst33342 (62249, Thermo Fisher, Waltham, MA, USA) staining was conducted to determine the presence of apoptosis.

### Caspase 3 activity assay

Caspase 3 activity was measured by using caspase-3 fluorescent assay kit (E13183, Thermo Fisher).

### Invasion assay

Invasion assay was performed by using Boyden chambers with a polycarbonate nucleopore membrane (Corning, Corning, NY, USA). Cells that migrated to the lower surface of the filter were fixed and stained with crystal violet and the cells counted in five random microscope fields per well. The extent of invasion was expressed as an average number of cells per microscope field.

### In vitro tube formation

HUVEC cells were then seeded on the Matrigel (356234, BD Bioscience)-coated wells, cultured at 37 °C in a 5% CO_2_ atmosphere incubator for 12–24 h, and observed with a light microscope equipped with a digital CCD camera to verify the formation of the capillary-like structures.

### Chick embryo chorioallantoic membrane (CAM) model of angiogenesis

This assay was performed in accordance with a previously published method [38]. Digital images of the CAM sections underneath the filters were collected, using a digital image analyzer (DMI-300, DMI, Korea). The number of vessel branch points contained in a circular region (equal to the area of the individual filter disk) was determined. One image was counted for each CAM preparation, and results from 6 to 8 CAM preparations were analyzed for each of the treatment conditions.

### Statistics

The statistical significance of differences was determined by GraphPad Prism8 (GraphPad, San Diego, CA). The results are expressed as mean ± SEM of the determinations. Groups were compared using one-way or tow-way ANOVA. Significance of the difference was accepted when the *p*-value was lower than 0.05.

## Supplementary information

**Additional file 1: Figure S1.** Effect of various inhibitors on the inhibition of their targets. (a) MDA-MB231 cells were pretreated with various inhibitors, PS-PKCζ (50 μM), Rottlerin (10 μM), AG1487 (10 μM), rapamycin (10 μM), B581 (50 μM), Bay117085 (5 μM), U0126 (20 μM), SP600125 (50 μM), SB203580 (20 μM), LY294002 (20 μM), PP2 (10 μM) for 30 min, and EGF (50 ng/mL) or PDGF (50 ng/mL) was treated for 10 min. The lysates were analyzed by western blot using the indicated antibodies. (b) For inhibitory effect of MTM, the cells were transfected with pSp1-Luc, and pretreated with MTM (5 μM) for 30 min, after which they were treated with PMA (50 nM) for 15 h. The luciferase activity was measured. Results are shown as the mean ± SEM. **p < 0.001.

## Data Availability

The analyzed datasets during present study are available from the corresponding author on reasonable request.

## Abbreviations

PLD2: Phospholipase D2
HDAC: Histone deacetylase
PA: Phosphatidic acid
SAHA: Suberoylanilide hydroxamic acid
TSA: Trichostatin A
CAM: Chick embryo chorioallantoic membrane
VEGF: Vascular endothelial growth factor
