# Supplementary figures and images for: Inhibition of phospholipase D2 augments histone deacetylase inhibitor-induced cell death in breast cancer cells
Source: Biol Res. 2020 Oct 1;53:34. doi: 10.1186/s40659-020-00294-3 (PMC7528251; doi:10.1186/s40659-020-00294-3)

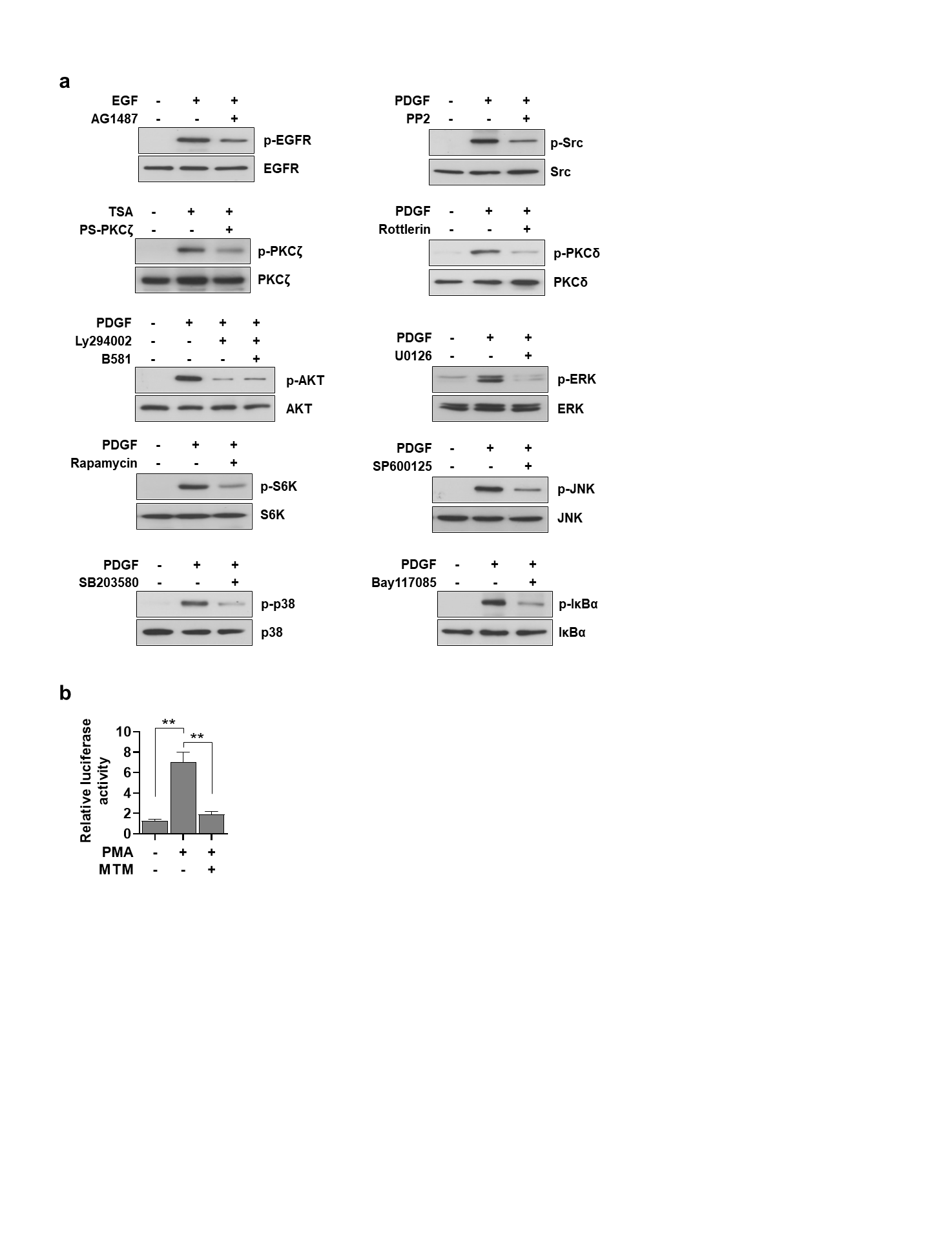

Supplement: Supplementary file 1 — Additional file 1: Figure S1. Effect of various inhibitors on the inhibition of their targets. (a) MDA-MB231 cells were pretreated with various inhibitors, PS-PKCζ (50 μM), Rottlerin (10 μM), AG1487 (10 μM), rapamycin (10 μM), B581 (50 μM), Bay117085 (5 μM), U0126 (20 μM), SP600125 (50 μM), SB203580 (20 μM), LY294002 (20 μM), PP2 (10 μM) for 30 min, and EGF (50 ng/mL) or PDGF (50 ng/mL) was treated for 10 min. The lysates were analyzed by western blot using the indicated antibodies. (b) For inhibitory effect of MTM, the cells were transfected with pSp1-Luc, and pretreated with MTM (5 μM) for 30 min, after which they were treated with PMA (50 nM) for 15 h. The luciferase activity was measured. Results are shown as the mean ± SEM. **p < 0.001. [file 40659_2020_294_MOESM1_ESM.tif]
